# In Vitro Antineoplastic and Antiviral Activity and In Vivo Toxicity of *Geum urbanum* L. Extracts

**DOI:** 10.3390/molecules27010245

**Published:** 2021-12-31

**Authors:** Maya M. Zaharieva, Lyudmila L. Dimitrova, Stanislav Philipov, Ivanka Nikolova, Neli Vilhelmova, Petar Grozdanov, Nadya Nikolova, Milena Popova, Vassya Bankova, Spiro M. Konstantinov, Dimitrina Zheleva-Dimitrova, Hristo M. Najdenski

**Affiliations:** 1 Department of Infectious Microbiology, The Stephan Angeloff Institute of Microbiology, Bulgarian Academy of Sciences, 26 Acad. G. Bonchev Street, 1113 Sofia, Bulgaria; zaharieva26@yahoo.com (M.M.Z.); lus22@abv.bg (L.L.D.); 2 Department of Human Anatomy, Histology, General and Clinical Pathology and Forensic Medicine, Faculty of Medicine, Sofia University St. Kliment Ohridski, 2 Kozyak Street, 1421 Sofia, Bulgaria; stanislav_philipov@abv.bg; 3 Department of Virology, The Stephan Angeloff Institute of Microbiology, Bulgarian Academy of Sciences, 26 Acad. G. Bonchev Street, 1113 Sofia, Bulgaria; vanianik@mail.bg (I.N.); nelivili@gmail.com (N.V.); nadyanik@yahoo.com (N.N.); 4 Laboratory Center Pasteur, The Stephan Angeloff Institute of Microbiology, Bulgarian Academy of Sciences, 26 Acad. G. Bonchev Street, 1113 Sofia, Bulgaria; grozdanov@microbio.bas.bg; 5 Laboratory Chemistry of Natural Products, Institute of Organic Chemistry with Centre of Phytochemistry, Bulgarian Academy of Sciences, 9 Acad. G. Bonchev Street, 1113 Sofia, Bulgaria; popova@orgchm.bas.bg (M.P.); bankova@orgchm.bas.bg (V.B.); 6 Department of Pharmacology, Pharmacotherapy and Toxicology, Faculty of Pharmacy, Medical University Sofia, 2 Dunav Street, 1000 Sofia, Bulgaria; Konstantinov.spiromihaylov@gmail.com; 7 Department of Pharmacognosy, Faculty of Pharmacy, Medical University Sofia, 2 Dunav Street, 1000 Sofia, Bulgaria; dimizheleva@gmail.com

**Keywords:** *Geum urbanum* L. extracts, in vitro antineoplastic and antiviral activity, UHPLC–HRMS analysis, acute in vivo toxicity

## Abstract

This study evaluated the in vitro antineoplastic and antiviral potential and in vivo toxicity of twelve extracts with different polarity obtained from the herbaceous perennial plant *Geum urbanum* L. (*Rosaceae*). In vitro cytotoxicity was determined by ISO 10993-5/2009 on bladder cancer, (T-24 and BC-3C), liver carcinoma (HEP-G2) and normal embryonic kidney (HEK-293) cell lines. The antineoplastic activity was elucidated through assays of cell clonogenicity, apoptosis induction, nuclear factor kappa B p65 (NFκB p65) activation and total glutathione levels. Neutral red uptake study was applied for antiviral activity. The most promising *G. urbanum* extract was analyzed by UHPLC–HRMS. The acute in vivo toxicity analysis was carried out following OEDC 423. The ethyl acetate extract of aerial parts (EtOAc-AP) exhibited the strongest antineoplastic activity on bladder cancer cell lines (IC_50_ = 21.33–25.28 µg/mL) by inducing apoptosis and inhibiting NFκB p65 and cell clonogenicity. EtOAc and *n*-butanol extracts showed moderate antiviral activity against human adenovirus type 5 and human simplex virus type I. Seventy four secondary metabolites (gallic and ellagic acid derivatives, phenolic acids, flavonoids, etc.) were identified in EtOAc-AP by UHPLC–HRMS. This extract induced no signs of acute toxicity in liver and kidney specimens of H-albino mice in doses up to 210 mg/kg. In conclusion, our study contributes substantially to the detailed pharmacological characterization of *G. urbanum*, thus helping the development of health-promoting phytopreparations.

## 1. Introduction

*Geum urbanum* L. (genus *Geum*, family *Rosaceae*) is a herbaceous perennial plant species, commonly known as wood avens or St. Benedict’s herb, and widely spread in the temperate regions of Europe [1,2], e.g., Bulgaria. It was used from ancient times in the European and particularly in the Bulgarian traditional medicine for gastro-intestinal disorders to reduce bleeding and the inflammation of mucous membranes [3,4,5]. The species is listed also in the VOLKSMED database as a traditional Austrian herb with anti-inflammatory activity [6]. Decoctions and infusions from aerial parts, roots and rhizomes were recommended for the treatment of diarrhea, dysentery, dyspepsia, gastroenteritis, leucorrhea, hemorrhages, infections and fever [4,6]. Modern scientific phytochemical analyses have proven that *Geum* species are rich in biologically active compounds, such as ellagitannins, procyanidines, phenolic acids, essential oils and other metabolites with different pharmacological effects [1,7,8], e.g., anti-inflammatory, antimicrobial and chemopreventive. The chemical composition of aerial and underground parts of different *Geum* species, as well as the antioxidant, anti-inflammatory and antibacterial properties of different *G. urbanum* extracts, has been well documented over the last decade [2,5,7,9,10,11]. Additionally, the antiviral potential of the species *G. japonicum* has been reported earlier; namely, herbal extracts from this species suppressed herpes simplex virus type 1 (HSV-1) as single treatment (EC_50_ = 52.8 μg/mL) or in combination with acyclovir (EC_50_ = 1.1 μg/mL) [12]. For the quercetin and luteolin found in *G. rivale* [13], *G. bulgaricum* [14,15] and *G. urbanum* [16], it was shown that, along with the antiviral activity, they exhibited significant antineoplastic effects [17]. The apigenin isolated from *G. rivale* [13] and *G. japonicum* [18] was reported to also show promising antineoplastic potential [19]. However, the information published to date about the in vitro and in vivo toxicity, antineoplastic and antiviral effects of *G. urbanum* extracts is still not comprehensive and needs to be expanded into more research [20,21].

Cancer is a heterogeneous disease involving a complex set of signs and symptoms where inflammatory and infectious diseases caused by bacteria and viruses play a significant role [22]. Various bacteria and viruses often affect the skin and mucosae, the digestive system, respiratory tract, the urinary and nervous system, etc. Numerous studies indicate that chronic infections and inflammation are associated with increased risk for cancer development [23,24]. Cytokines, chemokines, prostaglandins, and reactive oxygen and nitrogen radicals accumulate in the microenvironment of tissues affected by chronic inflammation. If persistent, these inflammatory factors have the capacity to induce cell proliferation, and promote cell survival through the activation of oncogene-suppressor or inactivation of tumor-suppressor genes, thus resulting in genetic instability with increased cancer risk [23,24]. Classic examples of the interrelationship between microbial infections, on one hand, and inflammation and carcinogenesis, on the other, are: (1) the role of *Helicobacter pylori* in the pathogenesis of gastric adenocarcinoma [25,26,27]; (2) the contribution of dento-gingival bacteria to the initiation and the promotion of oral squamous cell carcinoma [28]; (3) the association between staphylococcal infections and cutaneous T-cell lymphoma [29,30]; (4) persistent human papilloma virus (HPV) or herpes simplex type 2 virus (HSV-2) infections are associated with cervical cancer [31,32]; (5) increased risk for chemotherapy-induced oral mucositis in the presence of HSV-1 infection [33]; and many others. Other common neoplasms associated with infections are bladder carcinoma (the fourth most common tumor in women in Western countries and the seventh worldwide in men [34]), and liver carcinoma (the fifth most common neoplasia among men and ninth among women [35]). Clinical studies revealed that plant polyphenolic compounds exert antimicrobial, chemopreventive and antineoplastic effects in parallel [36], which are partially related to their antioxidant activity [37]. Therefore, the search for potential drug candidates which combine antioxidant, anti-inflammatory, antimicrobial and antineoplastic properties with a favorable toxicological profile is especially relevant nowadays. One possible approach is the exploitation of natural plant resources.

Taking into consideration the available information in the literature about the radical-scavenging, anti-inflammatory and antimicrobial effects of different *Geum* species and *G. urbanum* extracts, we aimed to evaluate for the first time the cytotoxicity, antineoplastic and antiviral activities of twelve extracts with different polarity according to the solvents used: six from aerial parts (AP) and six from underground parts (UP). The experimental design started with in vitro investigation of all extracts for cytotoxicity and continued with the evaluation of the antiviral and antineoplastic activities of the extracts with the lowest toxicity. For this aim, a panel of non-tumorigenic (human embryonic kidney) and malignant cell lines (bladder carcinoma and adenocarcinoma of the liver) and enveloped and non-enveloped viruses from four different families (*Picornaviridae*, *Adenoviridae*, *Paramixoviridae*, *Herpesviridae*) were chosen. The selected virus models are causative agents of socially important diseases in humans. Virus trains from the following five viral species were included—Poliovirus type 1 (PV-1), Coxsackie viruses B1 and B3 (CVB1, CVB3), human respiratory syncytial virus A2 (HRSV-A2), human adenovirus 5 (HAdV-5) and HSV-1. The tests for in vitro antiproliferative and pro-apoptotic effects in bladder carcinoma cell lines and the characterization of the acute in vivo toxicity on liver and kidney in H-albino mice were carried out only with the most potent extract, which showed the best selectivity index (SI) and the lowest value of the median inhibitory concentration (IC_50_) in tumor cell lines.

## 2. Results

### 2.1. Experimental Design

First, the evaluation of in vitro cytotoxicity on the non-tumorigenic cell lines HEK293 and HEP-G2 was carried out. The least toxic extracts were subjected to investigation of the above-mentioned pharmacological activities. We chose the cell lines HEK293 (normal transformed embryonal kidney cells) and HEP-G2 (hepatocellular carcinoma) for preliminarily evaluating the in vitro cytotoxicity of the investigated extract as a first step before performing the acute toxicity study, taking into consideration that the liver and kidney are the most important organs involved in the metabolism and excretion of xenobiotics after per oral administration. Although derived from a patient with hepatocellular carcinoma, the cell line HEP-G2 is often used as an in vitro model for hepatotoxicity, not only because it is non-tumorigenic (including in nude mice) like HEK293 but because it also has a high degree of morphological and functional differentiation. HEP-G2 cells perform many differentiated hepatic functions [38] and therefore cannot be described as being dedifferentiated—a hallmark of many malignant tumors. Furthermore, a preliminary test for antineoplastic activity was performed on the human tumor cell line T-24 originating from human bladder carcinoma, stage III. The most active EtOAc-AP extract (with the highest SI) was applied on a second bladder cancer cell line (BC-3C, stage IV), in order to confirm the results. The extract was further tested for in vitro cytotoxicity against normal mice fibroblasts (CCL-1 cell line). All obtained IC_50_ values were taken into consideration for the in vivo experiments based on the recommendations of Interagency Coordinating Committee on the Validation of Alternative Methods [39]. After the assessment of the extract’s ability to suppress malignant cell proliferation and to induce apoptosis, it was subjected to an acute in vivo toxicity study on H-albino mice. The general conditions of the test animals were described throughout the experiment and the liver and kidney tissues (usually involved in the metabolism and excretion of xenobiotics) were examined for pathomorphological changes. In parallel, a phytochemical analysis of the EtOAc-AP extract was performed.

### 2.2. In Vitro Cytotoxicity of G. urbanum Extracts on the Non-Tumorigenic Human Cell Line HEK293

The in vitro cytotoxicity test was performed with all twelve extracts on the non-tumorigenic cell line HEK293 for 72 h (Table 1). The petroleum ether (PET) and methanol (MeOH) extracts, as well as the EtOAc-AP extract, showed IC_50_ values lower than 100 µg/mL, and the PET-UP extract was the most cytotoxic. In contrast, the IC_50_ values of the *n*-butanol (*n*-BuOH), water (dH_2_O) and ethanol (EtOH) extracts ranged between 100 and 400 µg/mL, and the AP extracts were less cytotoxic than the UP extracts.

### 2.3. Antiproliferative Effects of EtOAc-AP Extract from G. urbanum in Tumor Cell Lines

The IC_50_ values after 72 h of incubation are given in Table 2. The SI for T-24 cells was calculated relative to the IC_50_ values determined for HEK293 cells. The highest SI was obtained for EtOAc-AP. The cytotoxic activity of the EtOAc-AP extract was confirmed by testing it on a second bladder cancer cell line (BC-3C) from a patient with stage IV of the disease. The calculated IC_50_ value was comparable to that determined for T-24 cells (Table 3). EtOAc-AP was about threefold less cytotoxic to the human liver cell line HEP-G2 (Table 3). HEP-G2 hepatocytes displayed a slightly lower sensitivity to EtOAc-AP than HEK293 fibroblasts. The IC_50_ values for this cell line, along with those obtained for HEK293, were used further for the determination of the test doses used in in vivo experiments for acute toxicity.

**Figure 1:**
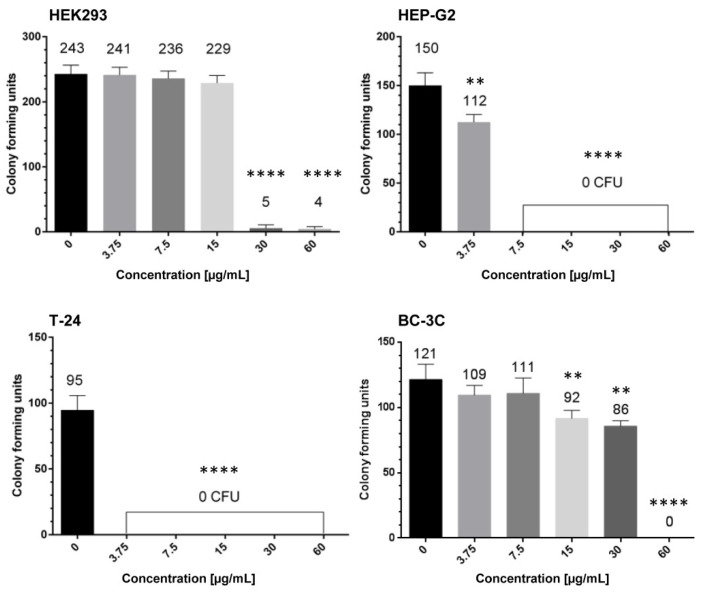
Clonogenicity of HEK293, T-24, BC-3C and HEP-G2 cells after treatment with EtOAc-AP extract of *G. urbanum*. The asterisks above the columns denote the *p*-value from the statistical analysis with one-way ANOVA obtained by comparison with the untreated control—** *p* < 0.01, **** *p* < 0.0001; the numbers above the columns represent the number of colonies.

Based on the SI values, we chose the most promising EtOAC-AP extract for further evaluation of its antineoplastic activity. Before starting the next assays, we also tested the in vitro cytotoxicity of this extract on the cell line CCL-1 which is recommended for such tests in Annex C of ISO 10993-5 [40]. The IC_50_ value for CCL-1 was slightly higher than that calculated for HEK293 cells and was 74.69 µg/mL (CI 95% = 66.98–83.30, m = −0.9, R = 0.98). The SI of EtOAC-AP based on CCL-1 cells was 2.95 for T-24 and 3.5 for BC-3C cells. Thereafter, the extract was evaluated for its potential to inhibit cell clonogenicity (thus predicting its long-term cytotoxicity) through colony-forming units assay (CFU assay). Data analysis from the CFU assay (Figure 1) showed that the colony formation of T-24 cells was inhibited up to 100% after exposure to concentrations of EtOAC-AP ≥ 3.75 µg/mL. The inhibition concentration for HEP-G2 cells was 7.5 µg/mL, while HEK293 and BC-3C were significantly less sensitive—the inhibitory concentrations that suppressed the cell clonogenicity fully were 30 and 60 µg/mL, respectively.

### 2.4. Pro-Apoptotic Effects of EtOAc-AP Extract of G. urbanum in Bladder Carcinoma Cell Lines

**Figure 2:**
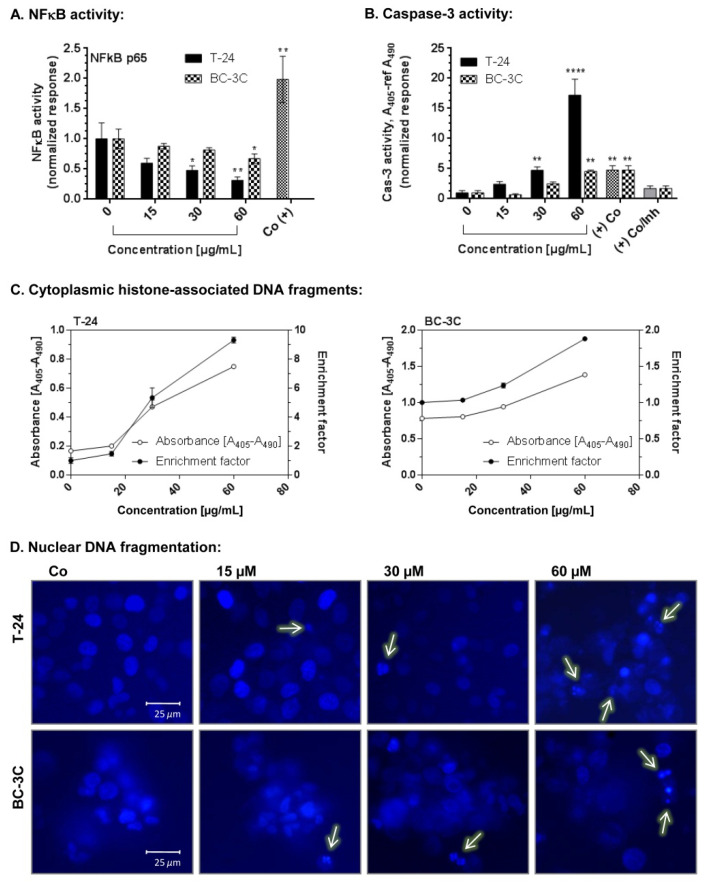
NFkB inhibition and induction of apoptosis in T-24 and BC-3C cell lines after treatment with EtOAc-AP extract of *G. urbanum*. The asterisks above the columns denote the *p*-value from the statistical analysis with one-way ANOVA obtained by comparison with the untreated control—* *p* < 0.05, ** *p* < 0.01, **** *p* < 0.0001; enrichment factor—normalization of the values against the untreated control assumed to be 1. Legend: (**A**)—activity of NF-κB p65 in T-24 and Bc-3C cells after exposure to three different concentrations of EtOAc-AP; (**B**)—activation of caspase 3 in T-24 and Bc-3C cells after exposure to three different concentrations of EtOAc-AP; (**C**)—accumulation of cytoplasmatic histone-associated DNA fragments in T-24 and Bc-3C cells after exposure to three different concentrations of EtOAc-AP; the two lines on each graph represent the absorbance (left *y*-axis) and the fraction of the untreated control (right *y*-axis); (**D**)—nuclear DNA fragmentation in T-24 and BC-3C cells after exposure to three different concentrations of EtOAc-AP; field magnification—400×.

Three different concentrations of EtOAc-AP extract (15, 30 and 60 µg/mL) were tested for the induction of programmed cell death in T-24 and BC-3C cells. The following markers of apoptosis were detected after a 48 h incubation period: activation of caspase-3, accumulation of cytoplasmic histone-associated DNA fragments and nuclear DNA fragmentation (Figure 2). A significant dose-dependent increase in the levels of activated caspase-3 was detected in both cell lines. This effect was more pronounced in the more sensitive cell line—T-24. Analyzing the data, it was found that caspase-3 levels in T-24 cells increased up to threefold after treatment with 60 μg/mL EtOAc-AP extract, compared to the untreated control, and that fact is a marker of apoptosis induction. Accordingly, a significantly higher amount of histone-associated DNA fragments was found in the cytoplasm of the treated T-24 cells than in the BC-3C cells. The nuclear DNA fragmentation analysis confirmed the results from the two other assays; namely, more cells with DNA fragments were observed under a fluorescent microscope after staining of the nuclei with Hoechst.

**Figure 3:**
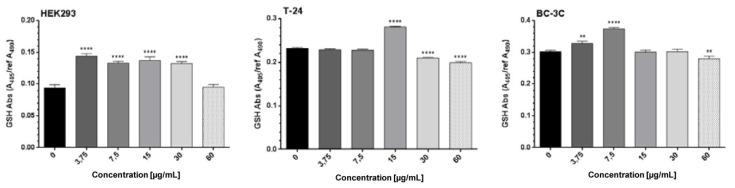
Reduction in GSH after treatment of HEK293, T-24 and BC-3C cell lines with EtOAc-AP extract of *G. urbanum*. The asterisks above the columns denote the *p*-value from the statistical analysis with one-way ANOVA obtained by comparison with the untreated control—** *p* < 0.01, **** *p* < 0.0001.

The results obtained regarding the total glutathione (GSH) levels (Figure 3) showed their small but significant decrease in BC-3C cells incubated with 60 µg/mL of the tested extract and in T-24 cells after treatment with 30 or 60 µg/mL extract. Lower EtOAc-AP concentrations (≤15 µg/mL) led to an increase or had no impact on the synthesis of GSH.

### 2.5. Antiviral Activity of Extracts from G. urbanum

The experimentally obtained data concerning the chemotherapeutic characteristics of the tested samples and referent compounds against the replication of PV1, CVB1, CVB3, HRSV-A2, HAdV-5 and HSV 1 are presented in Table 4. The tested extracts were found to vary in cytotoxicity from high to moderate levels. Their cytotoxic concentrations (CC_50_) in the HEp2 cell line were in the range between 39.7 and 167 µg/mL. The cytotoxicity concentrations (CC_50_) in the MDBK cell line are in range between 88.6 and 1200 µg/mL. *n*-BuOH-UP was the least toxic among all the tested samples. The strongest antiviral effect was observed in the cases of *n*-BuOH-AP tested against HSV 1 (SI 34) and *n*-BuOH-UP against HSV 1 (SI 19.4). Relatively the same selective indices were determined in the cases of EtOAc-UP tested against HAdV-5 (SI 18) and MeOH-UP against HSV 1 (SI 8.9). The rest of the extracts revealed a weak and rather marginal antiviral activity—EtOAc-AP against HAdV-5 (SI 5.8), and MeOH-AP against HSV 1 (SI 5.3). Each of the tested extracts did not reveal any antiviral activity against PV1, CVB3, and HRSV-A2.

### 2.6. UHPLC–HRMS Analysis of G. urbanum EtOAc-AP Extract

**Figure 4:**
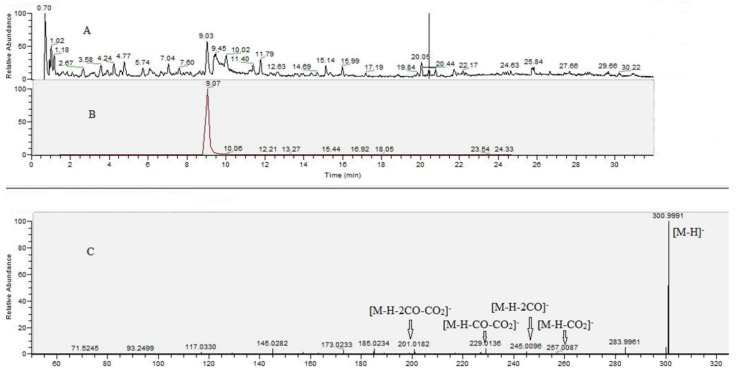
Total ion chromatogram in negative ion mode (**A**), extracted ion chromatogram of peak at *m*/*z* 300.9988 (**B**), MS/MS spectrum of peak at *m*/*z* 300.9988 (**C**).

The results from the UHPLC–HRMS analysis are presented in Figure 4 and Table 5. The total ion chromatograms of the *G. urbanum* EtOAc-AP extract are shown in Figure 4A. Based on comparison with reference standards, compounds **1** and **2** were identified as gallic and ellagic acid, respectively. The extracted ion chromatogram and MS/MS spectrum of the main compound (ellagic acid, **2**) in the extract are presented in Figure 4B,C. Gallic and ellagic acid derivatives (**3**–**11**, **14**–**21**) were identified based on the prominent ions at *m*/*z* 169.013 and *m*/*z* 300.999, matching the MS/MS fingerprint as **1** and **2**, respectively, and data published by [41,42,43,44]. Concerning **12** ([M-H]^−^ at *m*/*z* 477.068), the subsequent losses of hexose moiety (162.05 Da) at *m*/*z* 315.014 and methyl group (15.02 Da) at *m*/*z* 299.991 indicated a methylellagic acid derivative. Thus, **12** was identified as methylellagic acid *O*-hexoside (Table 4). Hydroxybenzoic and hydroxycinnamic acids (**23**–**34**), acylquinic acids (**35**–**48**), phenylethanoid glycosides (**49**–**56**) and flavonoids (**58**–**71**) were identified on the basis of the comparison with authentic standards and the literature data (Table 5).

In addition, two fatty and one triterpenoid acids were tentatively identified in the tested extract. Compound **72** yielded a deprotonated ion at *m*/*z* 187.096 (C_9_H_16_O_4_) together with the fragment ions at *m*/*z* 169.085 [M-H-H_2_O]^−^ and *m*/*z* 125.095 [M-H-H_2_O-CO_2_]^−^, suggesting carboxylic groups. This fragmentation pathway was previously described [45] and **72** was identified as azelaic acid. Concerning **73**, the base peak at *m*/*z* 183.138 ([M-H-CO_2_]^−^, supported by the diagnostic ion at *m*/*z* 165.127 [M-H-H_2_O-CO_2_]^−^, were indicative for the dicarboxylic, dodecenedioic acid (traumatic acid). The fragmentation pathway of 73 involved losses of 18 Da (H_2_O) at *m*/*z* 469.332, and 46 Da (H_2_O + CO) at *m*/*z* 423.327 together with the concomitant losses of 62 Da (H_2_O + CO_2_) at *m*/*z* 425.3414, suggesting triterpenoid acid with tertiary hydroxyl groups [46]. Accordingly, **73** was assigned as tormentic acid, previously isolated from *G. urbanum* roots [9].

### 2.7. Acute In Vivo Toxicity of EtOAc-AP Extract

#### 2.7.1. Common Signs of Acute Toxicity

Fourteen days after the administration of three different doses (Group I—210 mg/kg, Group II—70 mg/kg, Group III—20 mg/kg) of the *G. urbanum* EtOAc-AP extract, none of the treated animals showed any signs of toxicity. Mortality, body weight changes, and behavioral changes, e.g., reduced water and food consumption, were not observed. Liver and kidney tissue specimens were prepared and submitted to histopathological evaluation.

### 2.7.2. Pathomorphological Evaluation of Liver Tissue Specimens

**Figure 5:**
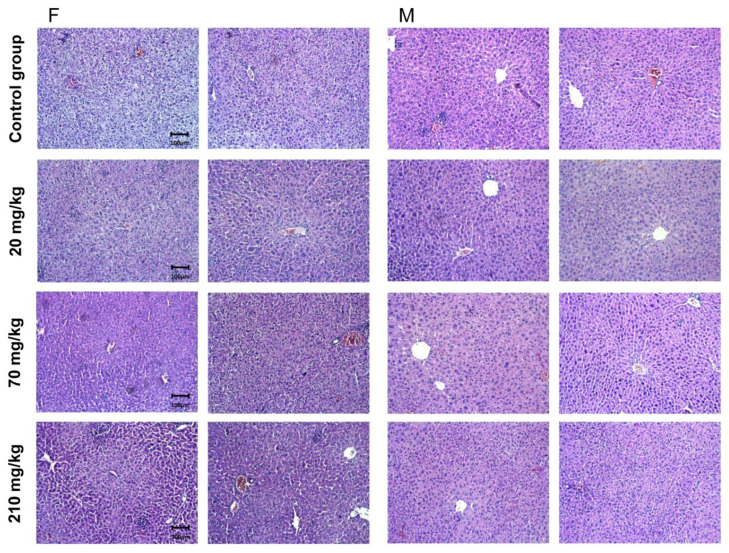
Pathomorphological findings of mice liver tissue specimens after oral administration of *G. urbanum* EtOAc-AP extract for 14 days. The four groups of animals with preparations from two animals of each sex and group are presented in the picture. Three groups of each sex were administered a different dose of the extract—20, 70 or 210 mg/kg. The control group received no extract. Legend: F—female animals; M—male animals; field magnification—200×.

Liver specimens of two of the tested male and female animals showed the isolated occurrence of individual changes (Figure 5). Pathological findings in the organs were not found. There was lobular recurrence in the parenchyma of the organs, a lack of remodeling zones, isolated intrahepatic cholestasis and regenerative activity. The findings were within tissue-specific parameters. Regenerative activity was detected in female animals with a very limited area. Bile systems included properly represented structures and a lack of portal canalicular proliferative changes. Circulatory changes in the organs included passive venous hyperemia, enlargement of sinusoidal spaces, and preserved hepatic lamellae. The large and middle veins of the organs were characterized by a well-preserved histological structure with no signs of changes in the vessel wall (intima and muscular layer). In female animals, the septal clones (equivalent to the terminal portal areas of the acina/acinus) were found not to traverse the entire distance between the two portal tracts. They were much thinner in the area of sinusoidal structures. Their location was in the middle of the interportal distances. They were not found in the spaces of the mall. Structural changes and dose dependence were not established. Isolated intrahepatic cholestasis was detected. Changes were absent in Group III organs (20 mg/kg) and isolated in organs from the other two study groups. Isolated intrahepatic cholestasis was detected in organs from male animals treated with 70 (Group II) and 210 mg/kg (Group I), but also with administration of 20 mg/kg (Group III). In female animals, changes were detected in Group I (210 mg/kg) but not in the other two groups. Hepatocyte loss was represented by individual cells and was not associated with a lobular inflammatory process and a proportional regenerative response. The absence of a lobular inflammatory process was accompanied by foci of portal and periportal inflammation. The outbreaks affected a small number of portal spaces and showed a limited area. Signs of minimal hepatocyte ballooning were identified. Changes were minimal for Group II and Group III. In Group I (210 mg/kg), low-level changes were observed with lobular ballooning varying in the narrow range (2–6%). In the female animals, changes were observed in the three study groups, with no increase in the number and area of outbreaks. Minimal steatosis (less than 5%) was found in both sexes and the representation was microvesicular. This type of change was found in all three experimental groups. The histological profile in male animals did not show an associated type of change—fatty degeneration of hepatocytes was of a non-zone type of distribution. Some cells showed cytoplasmic changes, centrally located nuclei, and isolated intracytoplasmic septa. The finding was not repeated and was not accompanied by the loss of membrane organelles. The putative metabolic overload of some types of membrane organelles was not accompanied by an increased turnover of these organelles. Autophagy vacuoles were observed as a single finding. In female animals, histological findings did not indicate an associated type of change—fatty degeneration was of a non-zonal type and metabolic zonation was of a gradient type. No loss of mitochondria, mitochondrial dysfunction, necrosis, and apoptosis was detected in any of the study groups.

#### 2.7.3. Pathomorphologial Evaluation of Kidney Tissue Specimens

**Figure 6:**
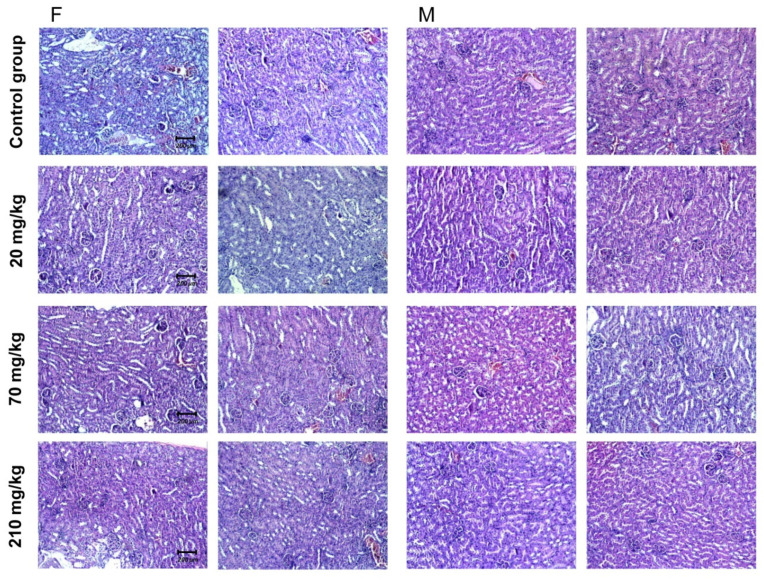
Pathomorphological findings of mice kidney tissue specimens after oral administration of *G. urbanum* EtOAc-AP extract for 14 days. The four groups of animals with preparations from two animals of each sex and group are presented in the picture. Three groups of each sex were administered with a different dose of the extract—20, 70 or 210 mg/kg. The control group received no extract. Legend: F—female animals; M—male animals; field magnification—200×.

Changes in female and male animals did not show organ polar topography: findings of two of the tested male and female mice are presented in Figure 6. The reported histological changes contain features consistent with normal histological architecture without being associated with concomitant pathological conditions. Vascular presentation was not accompanied by vascular fibro-intimal changes and luminal reduction. The cortical labyrinth was correctly represented with varying luminal zones in the proximal grooves at the height of the upholstery cells within the reference range. The bent portions of the distal tubules were characterized by a lower upholstery epithelium, wider luminal segments and preserved histological structure. Elements of glomerular and extraglomerular mesangium were visualized in separate areas and did not show signs of proliferation. Straight segments of proximal ducts were detected in isolated regions. The medullary part of the organs was marked by arched vessels. The outer area of the medullary part by the male animals differs in all studied groups. The outer and inner areas of the medullar part by female animals can be distinguished by much of the examined organs. The inner zone showed collecting ducts with the preserved histological structure. The findings did not show any differences in all three evaluated groups. The excretory sinus of the kidneys and the ampullary part of the renal pelvis had a preserved histological structure in all three studied groups. The histological findings are described in Table 6.

## 3. Discussion

In the present study, we evaluated for the first time the in vitro antineoplastic and antiviral potential of twelve AP and UP extracts with different polarity obtained from the medicinal plant *G. urbanum.* Thereafter, we selected the most promising, analyzed its phytochemical composition by UHPLC–HRMS and subjected it to a study for acute in vivo toxicity.

For evaluation of the antineoplastic activity of the extracts, we selected system bladder cancer cell lines as a model. Bladder carcinoma is the most common neoplasm of the urinary tract, with more new cases occurring in men but greater disease-specific mortality in women [47]. It is the fourth most common tumor in women in developed countries and the seventh worldwide in men [34]. In 2012, according to GLOBOCAN, 429 800 new and 165 100 lethal cases of bladder cancer were identified, making it the ninth most common cancer worldwide. However, the therapeutic schemes are not consistent, especially in the presence of metastases. The treatment includes radiotherapy and chemotherapy with phase-specific and phase-non-specific cytostatics. Particular attention is also paid to immune “checkpoint” inhibitors that have been developed since the 1980s [48]. A number of classic cytostatics that are used in the therapy of this cancer are of plant origin, such as paclitaxel. Therefore, the search for new natural compounds for therapy or concomitant treatment of this neoplasia is promising and deserves invested effort. In particular cases, the treatment could also be delivered locally, which widens the spectrum of anticancer agents used. Many extracts have shown promising potential for therapy of bladder carcinoma, including adjuvant intravesical treatment such as mistletoe (*Viscum album* L.) extract [49,50], EtOH extract of pomegranate [51], etc. All these data encouraged us to investigate the above-mentioned extracts for in vitro cytotoxicity in bladder cancer cell lines and to select the most suitable for further investigations. As a model for normal cell line, we chose normal human transformed embryonic kidney cells (HEK293).

Based on the IC_50_ values of the twelve tested extracts presented in Table 1, we can conclude that the EtOAc-AP extracts are less cytotoxic than the *n*-BuOH, dH_2_O and EtOH extracts. The differences in the cytotoxicity of the twelve tested extracts on HEK293 cells could be explained with the difference in the chemical composition of the extracts depending on the polarity of the solvent. Polar solvents (dH_2_O, 20% EtOH), solvents with intermediate polarity (MeOH, EtOH, *n*-BuOH, EtOAc, etc.) and solvents with low polarity (PET, *n*-hexane, CHCl_3_, etc.) are used to extract secondary metabolites from plants that differ in structure and polarity. Some authors mention that MeOH extracts contain tannins of low molecular weight, such as some hydrolysable tannins, and water can also react with them. Solvents with low polarity are often used to extract lipids and chlorophyll [52]. Therefore, extracts from the same plant material obtained with different solvents exhibit different biological properties [53].

In order to determine the antineoplastic activity of the extracts and to select the extracts with the highest SI for further investigations, we evaluated the in vitro cytotoxicity of all twelve extracts first on the tumor cell line T-24 (Table 2). As could be seen, the most active extract was EtOAc-AP, followed by the EtOAc-UP and PET-UP extracts. EtOAc-AP was characterized with the highest selectivity index, which is indicative of lower cytotoxic potential than the other extracts. Most probably, the high cytotoxic effect of EtOAc-AP extract in the tumor cell line is due to the high content of polyphenolic compounds (Table 5). The in vitro cytotoxicity of the EtOAc-AP was also tested on BC-3C, HEP-G2 and CCL-1 cells. As could be seen from the results based on the MTT assay, the tumorigenic cell line BC-3C was significantly more sensitive to exposure than the non-tumorigenic cells CCL-1 and HEP-G2.

Based on these results, we chose EtOAc-AP for investigation of its long-term antiproliferative properties by using the colony-forming units (CFU) assay. Its potential to inhibit cell clonogenicity was evaluated in bladder cancer, HEK-293 and HEP-G2 cells (Figure 1). The strong inhibition of the clonogenicity of T-24 cells revealed their high sensitivity to low concentrations of EtOAc-AP and proved the antiproliferative effect of the extract. In contrast, BC-3C cells were more resistant, requiring almost sixfold higher concentration for the inhibition of proliferation. This could be explained with the fact that this cell line originates from a patient with a more advanced stage of the disease (IV). Interestingly, the hepatocytes proved to be significantly more sensitive than BC-3C and HEK293 cells and lost their capacity to divide at a concentration six-fold lower than their IC_50_ value. A possible explanation is that the extract most probably suppressed important transduction pathways related to the malignant transformation of the hepatocyte cell line. It should be noted that although non-tumorigenic HEP-G2 cells originate from hepatocellular adenocarcinoma, it could be hypothesized that certain oncogenic transduction pathways which are activated in these cells are probably modulated after exposure to the extract, and this could be an object of detailed investigation in another study.

EtOAc-AP was also studied for its potential to induce apoptosis in T-24 and BC-3C cells. The results obtained show that EtOAc-AP extract exhibits antineoplastic activity in both bladder cancer cell lines because it does not only inhibit the cell proliferation as evidenced by MTT (Table 2 and Table 3) and CFU assays (Figure 1), but also induces apoptosis in a dose-dependent manner in concentrations near to the IC_50_ values (Figure 2). This effect of EtOAc-AP was expected because numerous publications about plants rich in ellagitannins and ellagic acid, such as *G. urbanum*, report that EtOAc, *n*-BuOH and MeOH extracts obtained from such plants are characterized with antiproliferative, anti-inflammatory and apoptosis-inducing activities [54]. One of the major targets of ellagitannis is nuclear factor kappa B (NF-κB) [55]. Inflammation in general and NF-κB in particular have a dual role in neoplasia. On one hand, the activation of NF-κB is part of the immune defense that targets and eliminates the transformed cells. This seems particularly true for acute inflammatory processes where the full activation of NF-κB is accompanied by high cytotoxic immune cell activity against cancer cells [56,57]. On the other hand, NF-κB is constitutively activated in many types of neoplasia and may exhibit pro-tumorigenic functions because the immune defense against malignant cells is not always effective enough to eliminate all aberrant cells, which in turn could gradually result in carcinogenesis [58]. The activation of NF-κB typically results in increased expression of anti-apoptotic genes, thereby providing a cellular survival mechanism that can withstand physiological stress, leading to an inflammatory response and, last but not least, to chemotherapy resistance [59,60]. Recently, Cui et al. [61] proved that NF-κB activation enhances the expression of survivin in bladder cancer cell lines and in vivo, leading thereby to enhanced proliferation and apoptosis inhibition. Particularly, the cell line T-24 expressed higher levels of survivin than the other investigated bladder cancer cell lines. Another study performed by Zhu et al. also revealed that NF-κB p65 overexpression promoted the migration of bladder cancer cells, thus mediating cell invasion and metastasis [62]. Our investigation shows that EtOAC-AP extract inhibits slightly but significantly the activation of NF-κB (Figure 2A), which supports the findings of [5] about the anti-inflammatory activity of *G. urbanum* extracts and is a promising result regarding the future development of the extract as a food additive with health-promoting benefits. The inhibition of NF-κB in T-24 cells was stronger than in the BC-3C cell line, which correlated with a stronger caspase 3 activity (Figure 2B) and higher accumulation of cytoplasmic histone-associated DNA fragments (Figure 2C). The greater inhibition of NF-κB p65 in the more sensitive cell line, T-24, leads to the suggestion that the apoptosis induction is related, at least in part, to the inhibition of NFκB p65. In BC-3C cells we observed a weaker inhibition of NF-κB. Respectively, the activation of caspase 3 and accumulation of cytoplasmic histone-associated DNA fragments in BC-3C cells were also weaker compared to T-24 cells. The BC-3C cell line is derived from a patient in a more advanced stage of the disease compared to T-24 and this could be a plausible explanation for the lower sensitivity of this cell line.

In vitro cytotoxic, antineoplastic and antiviral activities of plant extracts are generally related to the level of their antioxidant activity and the total polyphenolic content (TPC). The main reason for this effect is the fact that free radicals are mainly produced by oxidation processes and they contribute to human disorders such as aging-associated diseases, cardiovascular diseases, cancer and inflammatory infectious or non-infectious diseases [63]. Natural antioxidants found in many plant species help in controlling the formation of free radicals or prevent their interaction with biological molecules [64,65]. Consequently, due to scavenging of the free radicals, such compounds contribute to the antineoplastic and anti-inflammatory capacity of the plants containing them [66]. Polyphenols are well known for their anti-oxidant activities. Polyphenolic compounds increase the concentration of GSH and thereby alter cellular processes [67]. A decrease in cellular GSH levels has long been reported to be an early event in the apoptotic cascade induced by death receptor activation [68]. *Geum* species, including *G. urbanum*, are rich in polyphenols [9,10,11] and exhibit strong anti-oxidant properties. According to [10] the reported EtOAc-AP was characterized by stronger DPPH radical-scavenging activity than the *n*-BuOH and MeOH extracts, which was directly related to higher TPC. There are also published data about the relationship between antioxidant or anti-inflammatory activities of *G. urbanum* extracts and the measured TPC [5,9,10]. In line, the same trend for EtOAc-AP and other extracts was observed in our previous study [9], wherein the strong antioxidant activity and high TPC correlated with stronger antimicrobial activity. Considering the fact that the EtOAc-AP extract tested by us is characterized by a high content of phenols, as could be seen from our chemical analysis (Table 5) and from the published literature [9], we measured the concentration of total GSH in order to evaluate its role for the apoptosis induction in both bladder cancer cell lines after treatment (Figure 3). The slight but significant decrease in the total GSH levels in T-24 and BC-3C cell lines is indicative of a decrease in the cell reduction capacity and the abnormal generation of free radicals. It is clear from the results that the GSH regulation is most probably partially involved in the induction of apoptosis in the tested bladder cancer cell lines after exposure to 30 and 60 µg/mL of the extract, and the lower concentration is involved in GSH down-regulation only in the more sensitive T-24 cells.

The antiviral effect of *G. urbanum* was evaluated against viruses from different viral families (naked and enveloped viruses), that are important human pathogens. Six extracts from *G. urbanum* were tested against PV-1, CV–B1 and CV-B3 from the genus *Enterovirus* of *Picornaviridae*, HRSV-A2 from the *Paramyxoviridae*, HAdV-5 from *Adenoviridae*, and HSV-1 from the *Herpesviridae* family. Human enterovirus infections are distributed worldwide with extremely high morbidity, and although in most cases mild in their clinical course, they are manifested by a wide range of conditions and diseases including some severe illnesses of the central nervous system, the heart, endocrine pancreas, skeletal muscles, etc. No selective antiviral drug is registered to date for infections caused by enteroviruses and there are no specific drugs that can target adenovirus. Currently available drugs for the treatment of HSV-1 infection are the nucleoside analogs which prevent the production of proteins needed for viral replication [69]. The development of resistance to nucleoside analogs limits the options for treating viral infection, and that is why new alternative drugs are needed. The results obtained in our study reveal a promising property of *G. urbanum*—its antiviral capacity against HSV 1 and HAdV-5, for which chemotherapy is indicated.

The better understanding of the revealed biological activities of the EtOAc-AP extract tested in our study and the rationale for its mechanism of action depend on the detailed investigation of its chemical composition. Based on the accurate mass measurements, MS/MS fragmentation patterns, the relative abundance of the precursor and fragment ions, elemental compositions, monoisotopic peak profiles, and comparison with reference standards and literature data, 74 specialized natural products (21 gallic and ellagic acid derivatives, 12 hydroxybenzoic and hydroxycinnamic acids, 14 acylquinic acids, 8 phenylethanoid glycosides, 15 flavonoids and 3 others) were identified or tentatively elucidated in the most promising *G. urbanum* EtOAc-AP extract (Table 5). Our findings on specified natural products are in accordance with previously published data on *G. urbanum* extract [1,2,9]. The fact that the EtOAc-AP extract contains numerous polyphenolic compounds with well known anti-inflammatory and antineoplastic activities could explain its high antineoplastic potential and the presented mechanism of action.

The acute in vivo study revealed the good tolerability of the EtOAc-AP extract in H-albino mice, even in concentrations tenfold higher than the IC_50_ values, which were found to be effective during the in vitro experiments. The experimental animals showed no common signs of acute toxicity and no pathomorphological changes were found in the liver and kidney specimens. This result is promising for the further development of the extract as a potential food additive with a chemoprotective effect.

## 4. Materials and Methods

### 4.1. Plant Material

As described before, the dry aerial and underground parts from *G. urbanum* were provided by Sunny-Yambol, Ltd.^®^ (Yambol, Bulgaria) in April 2014 [9]. The extraction was performed immediately after the delivery of the products. All experiments were carried out with freshly prepared solutions of the dried extracts.

### 4.2. Chemicals and Reagents

The following media and buffers were used for the cell culture procedures: DMEM (Dulbecco’s Modified Eagle’s Medium, Cat. No. 12-604F, Lonza/BioWhittaker^®^, Walkersville, MD, USA), MEM (#MEM-A, Capricorn, Germany), horse serum (#HOS-1A, Capricorn, Germany), L-Glutamin 200 mM solution (Cat. No. TCL012-100ML, HiMedia^®^ Laboratories Pvt. Ltd., Mumbai, India), FBS (Fetal Bovine Serum, Cat. No. F7524, Sigma^®^ Life Science, Steinheim, Germany), and PBS (Dulbecco’s Phosphate-Buffered Saline, Cat. No. D8537, Sigma^®^ Life Science, Steinheim, Germany). The MTT dye [3-(4,5-dimethylthiazol-2-yl)-2,5-diphenyltetrazolium bromide] (Cat. No. M5655-1G, Sigma^®^Life Science, Steinheim, Germany) was dissolved in PBS to obtain 5 mg/mL solution and sterilized through filtration. The MTT solvent was prepared by adding 5% HCOOH (Chimspektar OOD, Sofia, Bulgaria) to absolute isopropyl alcohol (Cat. No. W292907-1KG-K, Sigma^®^Life Science, Steinheim, Germany). The methyl cellulose was purchased from Sigma^®^Life Science, Steinheim, Germany (Cat. No. M7027-100G) and a 2% sterile solution was prepared in RPMI-1640 medium (Cat. No. R1145, Sigma^®^Life Science, Steinheim, Germany) for the colony-forming units assay. The glutathione (Cat. No. G-6529, Sigma^®^Life Science, Steinheim, Germany) was applied for standard curve preparation. Ellmann’s reactive [5,5′-Dithiobis-(2-nitrobenzoic acid)] was purchased from Acros Organics (Geel, Belgium). The Hoechst (Sigma^®^Life Science, Steinheim, Germany) solution was prepared in a concentration of 100 mg/mL in distilled water (dH_2_O) and stored at −20 °C. For the extraction of plant material, the following chemicals were used: methanol (MeOH, 32213-M, Merck, Darmstadt, Germany), petroleum ether (24587, Honeywell, Charlotte, North Carolina, USA), ethyl acetate (EtOAc, 27227, Honeywell) and *n*-butanol (*n*-BuOH, 33065, Honeywell). Acetonitrile and formic acid for LC–MS and methanol of analytical grade were purchased from Merck (Merck, Sofia, Bulgaria). The reference standards used for compound identification were obtained as follows: salicylic acid, protocatechuic acid, gentisic acid, vanillic acid, ferulic acid, quercetin, luteolin, apigenin, kaempferol-3-*O*-glucoside, rutin, luteolin-7-*O*-glucoside, and chrysoeriol from Extrasynthese (Genay, France); ellagic acid, *m*-coumaric acid, *p*-coumaric, *o*-coumaric acid, caffeic acid, neochlorogenic (3-caffeoylquinic) acid, 3,4-dicaffeoylquinic acid, 1,5-dicaffeoylquinic acid were supplied from Phytolab (Vestenbergsgreuth, Germany); and chlorogenic acid was purchased from Sigma-Aldrich (St. Louis, MO, USA).

### 4.3. Preparation of G. urbanum Extracts

The extracts were prepared as previously described [9]. Briefly, 500 g underground and 500 g aerial parts of *G. urbanum* were macerated twice in 3 l MeOH at room temperature. The total MeOH extracts were filtered, concentrated in vacuo, and extracted successively with PET, EtOAc and *n*-BuOH. The aerial parts fractions were dried under vacuum to obtained 7.9 g PET, 10.4 g EtOAc and 17.82 g *n*-BuOH, and from underground parts: 1.9 g PET, 14.1 g EtOAc and 14.8 g *n*-BuOH. Part of the total MeOH extracts was evaporated to dryness and used in other experiments. A total of 10 g of underground and 10 g of aerial parts were extracted according to [70] with dH_2_O and 20% EtOH (32221-M, Merck) in an ultrasonic water bath at 50 °C for 1 h (1:40 *w*/*v*). The extracts were evaporated to dryness (for aerial parts: 2.16 g dH_2_O and 2.25 g 20% EtOH; for underground parts: 1.35 g dH_2_O and 0.94 g 20% EtOH). In general, twelve extracts were prepared for testing of their biological activities—six from aerial and six from underground parts, as follows: two MeOH (MeOH-AP, MeOH-UP), two PET (PET-AP, PET-UP), two *n*-BuOH (*n*-BuOH-AP, *n*-BuOH-UP), two EtOAc (EtOAc-AP, EtOAc-UP), two dH_2_O (dH_2_O-AP, dH_2_O-UP) and two water/ethanol (EtOH-AP, EtOH-UP) extracts.

### 4.4. Cell Lines and Culture Conditions

The in vitro cytotoxicity and antineoplastic activity of all *G. urbanum* extracts were tested on the non-tumorigenic cell line HEK293 (АСС-305, human embryonic kidney cells, Deutsche Sammlung fuer Mikroorganismen und Zelllinien DSMZ, Braunschweig, Germany), the bladder carcinoma cell lines T-24 (DSMZ ACC-376, disease stage III) and BC-3C (DSMZ ACC-450, disease stage IV), hepatocytes HEP-G2 (DSMZ АСС-180, adenocarcinoma of the liver stage I) and normal mice fibroblasts CCL-1^™^, NCTC clone 929 (L cell, L-929, derivative of Strain L) (American Type Culture Collection ATCC, Manassas, VA, USA). The cell lines HEp-2 (human epithelial type 2, HEp-2 CCL-23^™^) and MDBK (bovine kidney cells, MDBK (NBL-1) CCL-22^™^) originate from the ATCC and were used for growing the viral strains. The culture media were prepared according to the instructions of the bio banks providing the cell lines, namely DMEM supplemented with 10% FBS (HEK293, HEP-G2, T-24 and BC-3C cells) or 5% FBS (Hep-2 and MDBK cells), antibiotics (100 IU/mL penicillin, 100 μg/mL streptomycin, and 50 μg/mL gentamycin) and 20 mM HEPES buffer (Gibco BRL). CCL-1 cells were cultured in MEM with addition of 10% heat-inactivated horse serum and 2 mM L-glutamine. All cell lines were cultured at 37 °C in a humidified atmosphere, supplied with 5% CO_2_ (Panasonic CO_2_ incubator, #MCO-18AC-PE, Osaka, Japan) and routinely sub-cultured twice weekly. The experiments were carried out between the 5th and 10th passage of the cell lines.

### 4.5. Viruses

PV-1 (strain LSc-2ab) and CV-B1 and CV-B3 from the *Enterovirus* genus of the *Picornaviridae* virus family, HRSV-A2 from the *Paramyxoviridae* family, HAdV 5 from the *Adenoviridae* and HSV-1 from the *Herpesviridae* family were used for the antiviral tests. PV-1, CV-B1, CV-B3 and HRSV-A2 were grown in the Hep-2 cell line, and HSV-1 in MDBK cells. When harvesting viruses and performing antiviral assays, maintenance medium was used, in which the serum was reduced to 0.5%. Viruses were grown in a humidified atmosphere at 37 °C and 5% CO_2_.

### 4.6. In Vitro Cytotoxicity Tests

#### 4.6.1. MTT Test

The in vitro cytotoxicity of the *G. urbanum* extracts on HEK293, HEP-G2, T-24 and BC-3C cell lines was evaluated by the MTT reduction assay [71] with some modifications based on ISO 10993-5-2009, Annex C [40]. Briefly, cells were seeded in 96-well plates at density 7 × 10^3^ cells/well in 100 µL under sterile conditions (Laminar Air Flow Telstar Bio II Advance, Telstar, Terrassa, Spain). Thereafter, the plates were incubated for 24 h until cells reached 70% sub-confluence and the samples were treated with different extracts. The concentrations were applied as serial twofold dilutions from 0 up to 500 or 1000 µg/mL. Each concentration was prepared in quadruplicate repetition and the experiments were carried out in triplicate. After 72 h exposure to the extracts, the cell viability was determined with MTT solution (10 µL per sample). Plates were incubated for 3.5 h at 37 °C and the formazan crystals formed were dissolved with an equivalent volume of MTT solvent. The absorption of the samples was measured at λ = 550 nm (Absorbance Microplate Reader EL-800, Bio-Tek Instruments Inc., Winooski, VT, USA) against a blank solution (culture medium, MTT and solvent).

#### 4.6.2. Neutral Red Uptake Assay

The neutral red uptake assay based on the initial protocol described by Borenfreund and Puerner [72] and included in ISO 10993-5-2009, Annex A, was used for the evaluation of the in vitro cytotoxicity of three *G. urbanum* extracts on the cell lines HEp-2 and MDBK. Briefly, cells were incubated in 96-well plates using the appropriate culture medium. Monolayer cell cultures were inoculated with 0.1 mL/well maintenance medium containing different concentrations of the samples in 0.5–l g intervals. The controls consisted of cells incubated with DMEM only. After 48 h the culture medium containing the test compound was removed, cells were washed and 0.1 mL culture medium supplemented with 0.005% neutral red dye was added to each well. The plates were incubated at 37 °C for 3 h. After incubation, the neutral red dye was removed, cells were washed once with PBS and 0.15 mL/well desorb solution (1% glacial acetic acid, 49% ethanol, and 50% distilled water) was added. The optical density (OD) of each well was read at 540 nm in a microplate reader (Organon Teknika reader 530). The 50% cytotoxic concentration (CC_50_) was defined as the material concentration that reduced the cell viability by 50% when compared with untreated controls.

### 4.7. Determination of Antiviral Activity

Antiviral screening was based on the viral yield reduction technique. Monolayer cells in 96-well plates were inoculated with 0.1 mL virus suspension containing 100 CCID_50_ (cell culture infection dose 50%). The controls consisted of untreated infected cells, untreated non-infected cells (cell control) and the positive antiviral control (with referent compounds for each virus). After 1 h for virus adsorption, excessive virus was discarded, and cells were inoculated with 0.1 mL of maintenance medium containing different nontoxic concentrations (in 0.5–l g intervals) of the test samples. Then, cells were further incubated in a humidified atmosphere at 37 °C and 5% CO_2._ After 48 h the viable cells were stained according to the neutral red uptake procedure and the percentage of CPE (cytopathic effect) inhibition for each concentration of the test sample was calculated using the following formula: % CPE = [OD_test sample_ − OD_virus control_]/[OD_toxicity control_ − OD_virus control_] × 100, where OD_test sample_ is the mean value of the ODs of the wells inoculated with virus and treated with the test sample in the respective concentration, OD_virus control_ is the mean value of the ODs of the virus control wells (with no compound in the medium) and OD_toxicity control_ is the mean value of the ODs of the wells not inoculated with virus but treated with the corresponding concentration of the test sample. The IC_50_ was defined as the concentration of the material that inhibited 50% of viral replication when compared with the virus control. The SI was calculated from the ratio CC_50_/IC_50._

### 4.8. Colony-Forming Unit Assay

The colony forming unit (CFU) assay was applied to evaluate the clonogenicity of cells treated with 3.75, 7.5, 15, 30 and 60 µg/mL of *G. urbanum* EtOAc-AP extract. Briefly, after 48 h treatment, cells were trypsinized and counted and 10^3^ cells/mL from each sample were transferred into 5 mL of semi-solid medium (0.8% methylcellulose, 30% FBS). The suspension was plated into Petri dishes (1.2 mL/dish) with a diameter of 3500 mm and incubated for 7–10 days under standard cell culture conditions (37 °C, 5% CO_2_, humidified atmosphere). Colony formation (clusters of 20 or more cells) was scored under an inverted microscope. At least three samples per concentration were prepared. Data are represented as percentage of CFU [73,74,75].

### 4.9. Total GSH Assay

GSH levels were measured by a spectrophotometric method using Ellman’s reagent [76]. T-24 and BC-3C cells, treated with 15, 30 and 60 µg/mL EtOAc-AP extract, were trypsinized and counted in trypan blue; 5 × 10^5^ cells/sample were washed with PBS and centrifuged (400 g). The cell pellet was lysed in 100 µL 0.2M solution of EDTA in dH_2_O. Ice-cold trichloroacetic acid (20% *v*/*w*, 20 µL/sample) was added to precipitate the proteins. The volume was adjusted to 200 µL with dH_2_O, samples were centrifuged (14,500 rpm, 10 min) and the supernatant was analyzed for GSH in a 96-well plate, and then 160 µL TRIS buffer (0.4 M, pH 8.9) was mixed with 4 µL Ellman’s reagent (#117540010, Acros Organics, New Jersey, USA). The yellow product was measured at 410 nm (BioTek *EL* × 800). The intensity of absorbance is proportional to the amount of GSH. A calibration curve of 0.5–3.35 nmol GSH (#G6013, Merck, Darmstadt, Germany), dissolved in 0.2 M EDTA, was used as standard.

### 4.10. Hoechst Staining

DNA fragmentation was imaged by staining of live cells with Hoechst 33342 (#14533, Sigma^®^ Life Science). Briefly, cells were washed tree times with PBS, Hoechst solution in PBS (100 µg/mL final concentration) was added for 20 min [77] and the samples were observed on a Nikon Eclipse Ti-U CLSM using 20× plan apochromatic objective (final microscopic magnification 200×). The EZ-C1 software was used for image acquisition.

### 4.11. Caspase-3 Activity Assay

The Caspase-3 DEVD-R110 Fluorimetric & Colorimetric Assay Kit (#30008-1, Biotium, Fremont, CA, USA) was used for colorimetric measurement of the caspase-3 activity in cells treated with EtOAc-AP extract. Briefly, cells (T-24 and BC-3C) were treated with 15, 30 and 60 µg/mL extract for 24 h. Thereafter, 1 × 10^6^ cells were used for the assay following the instructions of the manufacturer. The absorbance of the product obtained by activated caspase-3 was measured at 495 nm (BioTek *EL* × 800). Cells incubated in hypertonic buffer (10 mM Tris, pH7.4, 400 mM NaCl, 5 mM CaCl_2_ and 10 mM MgCl_2_) for 2 h were used as a positive control. As a negative control, the competitive caspase-3 inhibitor Ac-DEVD-CHO (provided within the kit) was added to cells incubated in hypertonic buffer before the substrate incubation.

### 4.12. NFκB Assay

The activation of NF-κB in T-24 and BC-3C cells after treatment with EtOAc-AP was carried out using the commercial NFκB p65 (Total/Phospho) ELISA kit (#ADI-EKS-446, Enzo Life Sciences, Farmingdale, NY, USA) following the supplied protocol of the manufacturer. Briefly, treated and untreated cells were lysed with RIPA buffer (150 mM NaCl, 1% nonidet *p*-40, 0.5% sodium deoxycholate, 0.1% SDS, 25 mM Tris) and 10 µL of each was transferred into the ELISA microplate wells after incubation of the wells with working binding buffer. The plate with the strips was incubated at room temperature with mild agitation (300 rpm) for 1 h. Afterwards, the wells were washed three times with the kit wash buffer and subjected to immunoassay with primary NF-κB p65 antibody and secondary HRP-conjugated antibody. The signal of the active NF-κB p65 was detected using Luminol/Enhancer and stable peroxide solution on a c600 bio-analytical imaging system (Azure Biosystems, Inc., Dublin, CA, USA).

### 4.13. Ultra-High-Performace Liquid Chromatography—High-Resolution Mass Spectrometry (UHPLC–HRMS)

The most active extract (EtOAc-AP) was analyzed using UHPLC–HRMS. Mass analyses were carried out on a Q Exactive Plus mass spectrometer (ThermoFisher Scientific, Inc., Walthem, MS, USA) equipped with a heated electrospray ionization (HESI-II) probe (ThermoScientific). The tune parameters were as follows: spray voltage 3.5 kV; sheath gas flow rate 38; auxiliary gas flow rate 12; spare gas flow rate 0; capillary temperature 320 °C; probe heater temperature 320 °C; and S-lens RF level 50. Acquisition was acquired in full-scan MS and data-dependent MS^2^ modes. Full-scan spectra over the *m*/*z* range 100 to 1500 were acquired in negative ionization mode at a resolution of 70,000. Other instrument parameters for full MS mode were set as follows: AGC target 3e6, maximum ion time 100 ms, number of scan ranges 1. For DD-MS^2^ mode, instrument parameters were as follows: microscans 1, resolution 17,500, AGC target 1e5, maximum ion time 50 ms, MSX count 1, isolation window 2.0 *m*/*z*, stepped collision energy. Data acquisition and processing were carried out with Xcalibur 4.2 software (ThermoScientific).

### 4.14. Acute Toxicity Test

The acute toxicity of the most active extract (EtOAc-AP) was evaluated following the guideline OECD 423. Briefly, four groups of three male and three female H-albino mice were included in the experiment—one control group and three groups treated orally with different doses of the extract (Group I—210 mg/kg, Group II—70 mg/kg and Group III—20 mg/kg). The animal study was approved by the Animal Ethics Committee of the Bulgarian Food Safety Agency (Approval No. 125/07.10.2015-07.10.2020). Both sexes were included in the experiment because of a lack of knowledge about the toxicological or toxicokinetic properties of this extract in the literature. Mice of age 8–12 weeks weighing 21 ± 2 g were purchased from the National Breeding Centre (Slivnitza, Bulgaria) and housed in the Animal Care Facility of SAIM-BAS (352/06.01.2012, reg, No. 11130005), Bulgarian Academy of Sciences, in 12 h alternating light/dark cycles with free access to water and standard pelleted food ad libidum. They were kept in their cages for two weeks prior to dosing to allow acclimatization to the laboratory conditions. Before the experiment, each animal was marked to permit individual identification. The extract was dissolved in dH_2_O and solubilized by sonication and given orally once daily for fourteen days. Doses were prepared shortly prior to administration. The animals were observed daily for changes in weight, behavior and consumption of water and food. On the 14th day animals were euthanized humanely and the liver and the kidney were subjected to preparation of specimens for microscopic pathomorphological evaluation.

### 4.15. Histopathology

The paraffin sections prepared from liver and kidney tissue after 14 days oral treatment of the animals with EtOAc-AP extract (20, 70 and 210 µg/mL) were stained with hematoxylin and eosin (H&E) in order to demonstrate nucleus and cytoplasmic inclusions [78,79]. Hematoxylin precisely stains nuclear components in blue, including heterochromatin and nucleoli, while eosin stains cytoplasmic components, collagen and elastic fibers, muscle fibers and red blood cells in pink. Briefly, the paraffin sections were deparaffinized with xylene (a hydrocarbon solvent). The slides were passed through decreasing concentrations of EtOH—100%, 90%, 80%, 70%—and rinsed in water to remove the xylene and to be hydrated. Thereafter, they were stained for 5 min with filtered solution of the nuclear stain Harri’s hematoxylin (1 mg hematoxylin in 10 mL ethanol, 20 mg ammonium alum, boiled in distilled water and supplemented with 0.5 mg of mercuric oxide). After rinsing in tap water (5 min), the section was “blued” by treatment with a weakly alkaline solution. The non-specific background was removed using a weak acid alcohol (1% HCl in 70% alcohol for 5 min); the sections were washed in running tap water, dipped in an alkaline solution (e.g., ammonia water) and washed again with tap water. They were further stained with an aqueous solution of eosin (1 mg yellow eosin, 80 mL distilled water, 320 mL ethanol, 2 drops glacial acetic acid, 0.5% HCl) for 10 min and washed with tap water for 5 min. This colors many non-nuclear elements in different shades of pink. Following the eosin stain, the slides were dehydrated with increasing concentrations of ethanol, and rinsed in several baths of xylene in order to “clear” the tissue and render it completely transparent. A thin layer of polystyrene mounting was applied, followed by a glass cover slip.

### 4.16. Statistics

All in vitro experiments were performed in triplicate. The GraphPad Prism software (version 6.0.0 for Windows, San Diego, CA, USA) was applied for calculation of the IC_50_ values, preparation of all graphs and the statistical analysis of the data. The IC_50_ values of the tested substances from the MTT assay data were calculated based on the nonlinear regression model “*log(inhibitor) vs. normalized response, variable slope: Y = 100*/*(1 + 10^((LogIC50-X)×HillSlope))”.* The experimental data were analyzed statistically using the two-independent sample Student’s *t*-test and one-way ANOVA. Data are presented as the mean ± SD (standard deviation). A value of *p* < 0.05 was considered statistically significant.

## 5. Conclusions

In conclusion, our study contributes substantially to the detailed characterization of the pleiotropic pharmacological potential of *G. urbanum*, thus helping the further development of extracts thereof as health-promoting phytopreparations. The MeOH, *n*-BuOH and EtOAc extracts showed moderate to strong antiviral activity against different viral species. The EtOAc-AP extract of *G. urbanum* exhibits a promising antineoplastic activity in bladder carcinoma cells, expressed in the inhibition of their proliferation and the induction of programmed cell death. The extract is a rich source of ellagic acid, ellagitannins and other specialized natural products. Its favorable in vitro and in vivo toxicological profile in H-albino mice, characterized by a lack of signs of toxicity and pathological changes in the liver and kidney after two weeks of oral administration, is a good prerequisite for continuing research on its beneficial biological activities and mechanism of action.

## Figures and Tables

**Table 1:** In vitro cytotoxicity of *G. urbanum* extracts on the non-tumorigenic cell line HEK293 after 72 h of incubation.

Solvent	Part of the Plant	Parametersof the In Vitro Cytotoxicity Test			
		IC_50_ (µg/mL)	CI 95% *	m ^§^	R ^#^
Methanol	Aerial	58.30	53.6–63.4	−3.3	0.98
	Underground	58.52	57.1–59.9	−4.3	0.99
Ethyl acetate	Aerial	62.30	60.7–63.9	−7.3	0.99
	Underground	97.35	93.4–101.5	−5.3	0.99
*n*-Butanol	Aerial	141.6	132.1–151.8	−3.1	0.98
	Underground	115.6	108.7–122.9	−2.8	0.99
Petroleum ether	Aerial	72.72	65.1–81.2	−5.8	0.97
	Underground	40.25	37.2–43.5	−4.9	0.98
Water	Aerial	379.4	343.6–419.0	−7.9	0.98
	Underground	296.0	265.5–330.0	−3.8	0.96
20% Ethanol	Aerial	341.6	303.3–384.7	−1.9	0.95
	Underground	236.3	209.6–266.3	−1.9	0.96

Legend: *—95% confidence interval; ^§^—hillslope; ^#^—correlation coefficient.

**Table 2:** In vitro cytotoxicity of *G. urbanum* extracts on the tumor cell line T-24 after 72 h of incubation.

Solvent	Part of the Plant	Parameters of the In Vitro Cytotoxicity Test				
		IC_50_ (µg/mL)	CI 95% *	m ^§^	R ^#^	SI **
Methanol	Aerial	79.20	68.1–92.1	−1.5	0.95	0.74
	Underground	84.30	72.7–97.7	−0.9	0.96	0.69
Ethyl acetate	Aerial	25.28	24.1–26.5	−3.9	0.99	2.46
	Underground	42.09	39.3–45.1	−1.8	0.99	2.31
*n*-Butanol	Aerial	201.3	178.2–227.4	−1.2	0.97	0.70
	Underground	170.4	155.5–186.8	−1.5	0.98	0.68
Petroleum ether	Aerial	70.15	60.6–81.2	−1.6	0.95	0.83
	Underground	41.49	38.8–44.3	−2.4	0.99	0.97
Water	Aerial	338.4	293.4–390.3	−0.9	0.95	1.12
	Underground	295.4	267.1–326.7	−0.9	0.98	1.00
20% Ethanol	Aerial	323.5	253.9–412.1	−0.8	0.92	1.06
	Underground	343.4	281.0–419.6	−1.3	0.90	0.69

Legend: *—95% confidence interval; ^§^—hillslope; ^#^—correlation coefficient; **—index of selectivity (SI = IC_50_ of HEK293/IC_50_ of T-24).

**Table 3:** In vitro cytotoxicity of EtOAc-AP extract of *G. urbanum* on the tumor cell lines BC-3C and HEP-G2 after 72 h of incubation.

Parameters of the In Vitro Cytotoxicity Test	Cell Lines	
	BC-3C	HEP-G2
IC_50_ (µg/mL)	21.33	76.81
CI 95% *	18.4–24.7	68.9–85.5
m ^§^	−0.8	−1.4
R ^#^	0.97	0.98
SI **	2.92	0.81

Legend: *—95% confidence interval; ^§^—hillslope; ^#^—correlation coefficient; **—index of selectivity (SI = IC_50_ of HEK293/IC_50_ of BC-3C or HEP-G2).

**Table 4:** Antiviral activity of six *Geum urbanum* extracts.

Extracts	CytotoxicityCC_50_ [µg/mL]		PV1		CVB1		CVB3		HRSV-A2		HAdV-5		HSV 1	
	HEp 2	MDBK	IC_50_ *	SI	IC_50_	SI	IC_50_	SI	IC_50_	SI	IC_50_	SI	IC_50_	SI
n-BuOH-AP	167	1050	NA	-	NA	-	NA	-	NA	-	NA	-	54	19.4
n-BuOH-UP	63	962	NA	-	NA	-	NA	-	NA	-	NA	-	28.3	34
EtOAc-AP	58.5	88.6	NA	-	57	1.07	NA	-	NA	-	10	5.8	NA	-
EtOAc-UP	39.7	320	NA	-	NA	-	NA	-	NA	-	2.2	18	NA	-
MeOH-AP	50	220	NA	-	NA	-	NA	-	NA	-	NA	-	24.7	8.9
MeOH-UP	83.5	1200	NA	-	NA	-	NA	-	NA	-	30	2.7	224.5	5.3

Legend: *—μg/mL; NA—no activity; PV-1—poliovirus type 1; CVB1—Coxsackie B virus type 1; CVB3—Coxsackie B virus type 3; HRSV-A2—Human respiratory syncytial virus A2; HAdV-5—Human adenovirus type 5; HSV **1**—*Herpes simplex* virus type 1.

**Table 5:** Profile of secondary metabolites of *G. urbanum* (EtOAc-AP) by UHPLC–HRMS.

NO.	Identified/Tentatively Annotated Compound	Molecular Formula	Exact Mass [M-H]^−^	Fragmentation Pattern in (-) ESI-MS/MS	t_R_(min)	Δ ppm	Reference Standard (RS)/Reference
**Gallic and Ellagic Acid Derivatives**							
1.	gallic acid	C_7_H_6_O_5_	169.0132	169.0130 (34.67), 125.0228 (100), 97.0279 (3.25), 81.0331 (0.72), 69.0330 (4.70)	1.18	−7.257	RS
2.	ellagic acid	C_14_H_6_O_8_	300.9988	300.9991 (100), 245.0096 (1.81), 257.0087 (1.04), 229.0136 (3.67), 217.0139 (0.60), 201.0182 (3.83), 145.0282 (3.73), 117.0330 (1.04),	9.03	−0.500	RS
3.	gallic acid *O*-pentoside	C_13_H_16_O_9_	315.0728	315.0717 (7.32), 169.0131 (100), 125.0228 (39.01), 97.4442 (2.60)	4.77	2.205	Oszmiański et al., 2015
4.	galloylshikimic acid	C_14_H_14_O_9_	325.0565	325.0565 (46.17), 169.0131 (100), 137.0225 (4.63), 125.0228 (41.32), 111.0435 (8.16)	1.73	−0.047	Singh et al., 2015
5.	galloylglucose	C_13_H_16_O_10_	331.0673	331.0673 (100), 271.0462 (14.20), 211.0241 (16.18), 169.0130 (71.64), 151.0021 (3.93), 125.0229 (38.49), 125.0229 (38.49), 113.0227 (2.62), 89.0229 (2.82), 71.0121 (4.67), 59.0122 (3.84)	0.75	−2.130	Singh et al., 2015
6.	galloylglucose isomer	C_13_H_16_O_10_	331.0674	331.0674 (57.45), 271.0460 (87.40), 241.0351 (37.09), 211.0239 (30.67), 169.0130 (100), 161.0442 (5.55), 125.0228 (47.72), 107.0122 (7.29)	1.13	1.027	Singh et al., 2015
7.	ellagic acid *O*-pentoside	C_14_H_6_O_8_	433.0410	433.0413 (100), 300.9991 (67.43), 299.9912 (54.22), 245.0091 (0.82), 257.0091 (0.62), 229.0145 (2.03), 201.0199 (0.97)	8.60	−0.460	Oszmiański et al., 2015
8.	ellagic acid *O*-deoxyhexoside	C_20_H_16_O_12_	447.0569	447.0577 (100), 300.9992 (66.28), 299.9915 (85.37)	8.81	−0.110	Oszmiański et al., 2015
9.	ellagic acid *O*-hexoside	C_20_H_16_O_13_	463.0524	463.0525 (100), 300.9994 (67.48), 299.9904 (27.14)	6.80	2.451	
10.	flavogallonic acid	C_21_H_10_O_13_	469.0029	469.0029 (10.53), 425.0157 (100), 299.9918 (92.60), 298.9836 (26.35), 135.2071 (4.21)	6.21	−4.123	Singh et al., 2015
11.	ellagic acid *O*-hexuronide	C_20_H_14_O_16_	477.0312	477.0312 (57.99), 446.6355 (2.70), 300.9991 (100), 229.0140 (4.54), 244.5380 (3.12), 299.9882 (5.27)	6.54	1.321	
12.	methylellagic acid *O*-hexoside	C_21_H_18_O_13_	477.0685	477.0685 (100), 315.0148 (72.30), 299.9916 (54.33), 298.9822 (8.02), 270.9877 (15.75)	9.18	3.406	
13.	HHDP	C_20_H_18_O_14_	481.0628	481.0628 (100), 462.4490 (1.07), 300.9991 (77.43), 275.0194 (37.30), 229.0132 (8.40), 201.0191 (5.68), 185.0240 (1.90)	0.75	0.960	Singh et al., 2015
14.	HHDP isomer	C_20_H_18_O_14_	481.0628	481.0628 (100), 421.0443 (100), 300.9991 (69.36), 275.0194 (37.91), 229.0138 (8.30), 201.0188 (5.03), 185.0230 (2.20)	0.75	0.960	Singh et al., 2015
15.	digalloylglucose	C_20_H_20_O_14_	483.0748	483.0748 (100), 331.0676 (15.47), 313.0550 (9.74), 211.0246 (2.04), 169.0130 (82.73), 125.0229 (48.03), 151.0027 (2.15), 107.0123 (5.89)	1.67	0.831	Singh et al., 2015
16.	digalloylglucose isomer	C_20_H_20_O_14_	483.0785	483.0785 (100), 331.0676 (4.70), 313.0576 (13.31), 271.0459 (45.44), 211.0246 (11.17), 169.0130 (36.40), 125.0230 (32.87), 107.0122 (5.92)	4.23	0.893	Singh et al., 2015
17.	gemin D	C_27_H_22_O_18_	633.0740	633.0740 (100), 613.0474 (18.37), 481.0623 (3.77), 465.0675 (20.40), 445.0409 (8.60), 421.0831 (0.58), 319.0095 (4.01), 313.0565 (22.78), 300.9990 (54.64), 299.9922 (3.36), 275.0194 (7.77), 29.0134 (5.64), 245.0084 (2.45), 217.0132 (2.27), 125.0229 (23.25)	3.73	1.000	
18.	pedunculaginn	C_34_H_24_O_22_	783.0676	783.0676 (79.16), 688.7011 (1.95), 481.0623 (2.05), 342.8359 (1.81), 300.9991 (100), 275.0196 (38.55), 229.0132 (15.14), 203.0343 (2.92), 201.187 (8.05), 185.0237 (7.04), 245.0083 (3.19), 145.0277 (2.78)	1.85	−1.373	Hager et al., 2008
19.	pedunculagin isomer	C_34_H_24_O_22_	783.0696	783.0696 (94.52), 696.1905 (3.00), 632.2634 (2.81), 578.0688 (2.56), 419.6635 (3.15), 300.9991 (100), 275.0197 (53.08), 257.0084 (10.56), 229.0132 (15.28), 203.0343 (5.70), 201.187 (6.12), 185.0230 (2.82)	3.28	1.894	Hager et al., 2008
20.	tellimagrandin I	C_34_H_26_O_22_	785.0858	785.0858 (100), 492.5254 (2.38), 300.9992 (91.82), 275.0194 (40.60), 249.0403 (41.84), 229.0137 (14.38), 185.0232 (4.49), 169.0126 (13.49)	4.15	1.853	Singh et al., 2015
21.	galloyl-bis-hexahydroxyphenoyl-hexoside (casuarictin/ potentillin)	C_41_H_28_O_26_	935.0792	935.0792 (100), 633.0712 (2.11), 300.9989 (81.10), 229.0136 (7.32), 245.0079 (2.49), 257.0091 (4.42), 217.0138 (2.68)	9.46	−0.475	Donno et al., 2013
22.	trisgalloyl HHDP glucose	C_41_H_28_O_27_	951.0797	951.0793 (30.06), 907.0871 (100), 847.7051 (3.06), 783.0707 (6.71), 635.1165 (3.83), 408.0540 (3.33), 341.2052 (3.05), 299.9902 (26.04), 300.9986 (85.18), 275.0205 (28.77), 245.0072 (5.83), 229.0132 (5.75), 257.0094 (5.38), 201.0178 (7.20)	3.19	−0.717	Singh et al., 2015
**Hydroxybenzoic and hydroxycinnamic acids**							
23.	salcylic acid	C_7_H_6_O_3_	137.0230	137.0229 (14.31), 93.0329 (100), 65.0381 (0.53)	10.10	−0.271	RS
24.	protocatechuic acid	C_7_H_6_O_4_	153.0181	153.0180 (15.10), 123.0438 (1.17), 109.0279 (100)	2.04	−7.855	RS
25.	2,4-dihydroxybenzoic acid	C_7_H_6_O_4_	153.0181	153.0180 (15.10), 123.0438 (1.17), 109.0279 (100)	3.56	−8.770	
26.	gentisic acid	C_7_H_6_O_4_	153.0180	153.0180 (41.70), 123.0071 (0.23) 109.0279 (100)	4.71	−8.770	RS
27.	*p*-coumaric acid	C_9_H_8_O_3_	163.0387	163.0389 (87.03), 135.0436 (96.46), 119.0487 (100)	2.955	−8.510	RS
28.	*m*-coumaric acid	C_9_H_8_O_3_	163.0387	163.0387 (40.10), 135.0436 (30.46), 119.0487 (100)	3.92	−8.142	RS
29.	*o*-coumaric acid	C_9_H_8_O_3_	163.0390	163.0389 (7.89), 119.0486 (100)	6.87	−7.222	RS
30.	isovanillic acid	C_8_H_8_O_4_	167.0342	167.0342 (15.00), 152.0102 (100), 124.0147 (2.04)	4.26	−4.622	
31.	vanillic acid	C_8_H_8_O_4_	167.0342	167.0338 (100), 152.0098 (33.78), 124.0147 (11.99), 111.0070 (5.05), 95.0123 (3.62)	7.12	−4.921	RS
32.	caffeic acid	C_9_H_8_O_4_	179.0340	179.0340 (17.92), 135.0437 (100), 107.0488 (1.45)	4.77	−5.764	RS
33.	ferulic acid	C_10_H_10_O_4_	193.0500	193.0500 (8.88), 178.0263 (1.96), 149.0593 (3.56), 134.0359 (100)	8.65	−3.481	RS
34.	isoferulic acid	C_10_H_10_O_4_	193.0496	193.0496 (100), 178.0268 (6.84), 161.0231 (18.57), 149.0586 (2.18), 134.0360 (10.60)	11.63	−3.860	
**Acylquinic acids**							
35.	3-*p*-coumaroylquinic acid	C_16_H_18_O_8_	337.0925	337.0925 (13.04), 191.0550 (16.15), 173.0443 (3.40), 163.0387 (100)	3.97	0.651	Clifford et al., 2005
36.	1-*p*-coumaroylquinic acid	C_16_H_18_O_8_	337.0936	337.0936 (10.27), 191.0551 (100), 173.0446 (6.66), 163.0390 (7.41)	6.04	2.015	Clifford et al., 2005
37.	4-*p*-coumaroylquinic acid	C_16_H_18_O_8_	337.0931	337.0931 (8.57), 191.0551 (2.09), 173.0443 (100), 163.0387 (18.30)	6.35	0.562	Clifford et al., 2005
38.	5-*p*-coumaroylquinic acid	C_16_H_18_O_8_	337.0918	337.0918 (8.80), 191.0550 (100), 163.0389 (15.75)	7.72	−3.236	Clifford et al., 2005
39.	1-caffeoylquinic acid	C_16_H_18_O_9_	353.0876	353.0876 (72.68), 191.0550 (100), 179.0341 (83.65), 135.0437 (77.23)	2.08	−0.440	Clifford et al., 2005
40.	neochlorogenic acid	C_16_H_18_O_9_	353.0880	353.0881 (46.28), 191.0551 (100), 179.0339 (65.78), 173.0446 (4.13), 135.0437 (54.34)	2.64	0.495	RS
41.	chlorogenic acid	C_16_H_18_O_9_	353.0870	353.0887 (4.75), 191.0551 (100), 179.0335 (1.68), 161.0231 (2.06), 135.0438 (1.84)	4.34	2.676	RS
42.	4-caffeoylquinic acid	C_16_H_18_O_9_	353.0880	353.0881 (31.42), 191.0552 (40.86), 179.0341 (65.74), 173.0444 (100), 135.0437 (50.91)	4.84	0.410	Clifford et al., 2005
43.	3-feruloylquinic acid	C_17_H_20_O_9_	367.1041	367.1032 (24.57), 193.0497 (100), 173.0452 (2.42), 134.0359 (58.04)	4.79	1.756	Clifford et al., 2005
44.	4-feruloylquinic acid	C_17_H_20_O_9_	367.1042	367.1036 (12.06), 173.0444 (100), 163.5176 (7.37), 134.0361 (7.81)	6.68	2.083	Clifford et al., 2005
45.	5-feruloylquinic acid	C_17_H_20_O_9_	367.1045	367.1018 (17.78), 191.0551 (100), 173.0447 (8.89), 134.0358 (7.59)	7.30	2.737	Clifford et al., 2005
46.	1,5-dicaffeoylquinic acid	C_25_H_24_O_12_	515.1214	515.1190 (18.05), 353.0888 (86.26), 19.0551 (100), 179.0333 (44.18), 135.0434 (37.39)	11.42	3.690	Clifford et al., 2007
47.	3,5-dicaffeoylquinic acid	C_25_H_24_O_12_	515.1214	515.1217 (24.72), 353.0873 (77.33), 191.0555 (100), 179.0331 (29.88), 135.0437 (33.09)	11.79	3.690	Clifford et al., 2007
48.	4,5-dicaffeoylquinic acid	C_25_H_24_O_12_	515.1215	353.0865 (39.29), 191.0551 (35.74), 179.0340 (59.64), 173.0447 (100), 135.0435 (45.53)	12.63	3.923	Clifford et al., 2007
**Phenylethanoid glycosides**							
49.	protocatechuic acid *O*-hexoside	C_13_H_16_O_9_	315.0732	314.9041 (2.36), 153.0180 (100), 109.0279 (53.45)	1.24	3.348	
50.	coumaroyl hexose	C_15_H_18_O_8_	325.0922	325.0922 (6.89), 265.0716 (100), 235.0608 (42.67), 205.0498 (67.25), 163.0388 (67.25), 145.0280 (76.94), 119.0486 (54.37)	5.02	−2.155	
51.	*O*-caffeoyl hexose	C_15_H_18_O_9_	341.0878	341.0878 (29.75), 281.0663 (1.28), 251.0565 (2.69), 179.0338 (34.12), 161.0231 (100), 135.0437 (15.68), 133.0280 (26.58), 119.0335 (0.68)	3.22	−0.016	Clifford et al., 2007
52.	caffeic acid *O*-hexoside	C_15_H_18_O_9_	341.0877	341.0880 (20.12), 281.0666 (96.48), 251.0558 (51.86), 221.0450 (51.79), 179.0338 (100), 161.0231 (68.89), 135.0437 (65.22), 133.0280 (22.11), 119.0331 (1.41)	3.57	−0.367	Clifford et al., 2007
53.	caffeic acid *O*-hexoside isomer	C_15_H_18_O_9_	341.0877	341.0878 (26.20), 281.0667 (99.55), 251.0560 (58.13), 221.0448 (58.13), 179.0339 (100), 161.0231 (61.33), 135.0438 (78.37), 133.0281 (24.21)	4.27	−0.191	Clifford et al., 2007
54.	*O*-caffeoyldihexose	C_21_H_28_O_14_	503.1422	503.1417 (100), 323.0759 (4.92), 179.0339 (28.23), 161.0231 (64.90), 135.0437 (23.88), 133.0284 (16.6)	4.51	3.143	Oszmiański et al., 2015
55.	dicaffeoylhexose	C_24_H_24_O_12_	503.1220	503.1220 (87.04), 323.0788 (8.57), 179.0340 (100), 161.0229 (47.85), 135.0435 (87.65), 133.0283 (18.14)	11.20	4.931	
56.	dicaffeoylhexose isomer	C_24_H_24_O_12_	503.1207	503.1204 (100), 323.0760 (12.05), 179.0341 (70.79), 161.0229 (45.51), 135.0437 (84.87), 133.0277 (11.39)	12.69	3.477	
**Flavonoids**							
57.	apigenin	C_15_H_10_O_5_	269.0455	269.0455 (100), 151.0020 (5.22), 149.0230 (6.31)	17.92	−0.285	RS
58.	luteolin	C_15_H_10_O_6_	285.0410	285.0410 (100), 241.0148 (1.07), 201.0184 (1.46), 151.0022 (1.01), 133.0280 (4.64), 107.0122 (0.39)	15.37	1.785	RS
59.	chrysoeriol	C_16_H_12_O_6_	299.0558	299.0558 (55.66), 284.0324 (100), 255.0298 (34.04), 227.0349 (23.67)	19.77	−0.974	RS
60.	isorhamnetin	C_16_H_12_O_7_	315.0520	315.0513 (100), 300.0270 (41.20), 227.1276 (2.72), 151.0016 (2.86), 107.0123 (5.43)	19.18	3.695	RS
61.	homoorientin	C_21_H_20_O_11_	447.0946	447.0940 (100), 357.0617 (37.28), 327.0516 (54.46), 299.0566 (10.01), 297.0417 (7.40),	8.17	3.032	RS
62.	orientin	C_21_H_20_O_11_	447.0941	447.0941 (77.13), 357.0606 (38.10), 327.0519 (100), 297.0399 (7.35)	8.43	1.801	RS
63.	kaempferol 3-*O*-glucoside	C_21_H_20_O_11_	447.0957	447.0957 (100), 285.0403 (19.61), 284.0327 (53.17), 255.0297 (47.69), 227.0344 (47.98), 211.0408 (0.93), 151.0023 (1.08), 107.0119 (1.01)	11.42	5.402	RS
64.	isorhamnetin 3-*O*-pentoside	C_21_H_20_O_11_	447.0944	447.0455 (100), 366.6548 (2.12), 315.0150 (73.08), 314.0061 (17.27), 299.9912 (63.49), 285.0406 (60.99), 284.0327 (26.08), 270.9878 (15.03), 151.0026 (1.82),	10.28	2.405	de Rijke et al., 2006
65.	luteolin 7-*O*-glucoside	C_21_H_20_O_11_	447.0947	447.0947 (100), 285.0408 (56.48), 257.0452 (3.75), 151.0020 (27.49)	11.77	3.233	RS
66.	isoquercitrin	C_21_H_20_O_12_	463.0888	463.0888 (100), 301.0356 (31.89), 300.0275 (66.48), 271.0246 (33.03), 255.0304 (11.37), 243.0298 (7.18), 227.0348 (2.34), 151.0024 (1.83)	9.98	1.319	RS
67.	quercetin−7-*O*-hexoside	C_21_H_20_O_12_	463.0896	463.0896 (88.80), 301.0355 (100), 151.0023 (37.18), 107.0122 (15.76)	13.12	3.025	
68.	quercetin 3-O-hexuronide	C_21_H_18_O_13_	477.0592	477.0592 (57.61), 301.0361 (100), 178.9989 (7.63), 151.0019 (19.23)	9.74	−0.116	Oszmiański et al., 2015
69.	kaempferol 3-*O*-rutinoside	C_27_H_30_O_15_	593.1308	593.1308 (100), 285.0403 (68.74), 284.0326 (56.73), 255.0296 (34.96), 227.0345 (22.63), 107.0125 (1.80)	2.759	16.43	RS
70.	rutin	C_27_H_30_O_16_	609.1484	609.1484 (100), 301.0356 (35.08), 300.0284 (54.53), 271.0243 (28.18), 255.0314 (7.49)	9.60	4.630	RS
71.	luteolin 3-*O*-caffeoylhexoside	C_30_H_26_O_14_	609.1263	609.1263 (80.32), 285.0402 (100), 161.0233 (15.83), 135.0441 (4.12), 133.0275 (3.93), 151.0033 (3.49)	16.12	2.235	
**Others**							
72.	azelaic acid	C_9_H_16_O_4_	187.0968	187.0966 (40.44), 169.0859 (0.91), 163.5142 (0.67), 125.0957 (100), 97.0642 (8.67)	11.83	−1.464	Zhao et al., 2016
73.	traumatic acid	C_12_H_20_O_4_	227.1285	227.1285 (10.51), 209.1178 (1.02), 183.1379 (100), 165.1273 (18.23), 81.1410 (0.53)	20.50	−1.639	Sinan et al., 2020
74.	tormentic acid	C_30_H_48_O_5_	487.3419	487.3436 (100), 469.3323 (8.83), 425. 3414 (), 423.3272 (1.35), 379.2236 (0.33), 96.0703 (0.38)	25.76	−2.130	

**Table 6:** Histological findings in kidney tissue specimens of female and male mice with EtOAc-AP extract of *G. urbanum* administrated orally for 14 days.

Histological Findings *	Group I—210 mg/kg	Group II—70 mg/kg	Group III—20 mg/kg
Tubulitis—outbreaks of 5–10 cells per tubular diameter	Not established		
Mononuclear interstitial inflammatory infiltrate: less than 10% in female animals	8%	6%	4%
Mononuclear interstitial inflammatory infiltrate: less than 10% in male animals	6%	6%	3%
Glomerulitis—segmental and global in the presented glomeruli	Not established		
PAC-positive hyaline thickening in more than one arteriolus	Arterial patency was reported.		
Intimate arteritis with loss of luminal spaces in each arterial irrigation zone	No changes and luminal reduction in irrigation zones		
Infarcts	Not established		
Interstitial bleedings	Not established		
Glomerulopathic changes—double contouring peripheral capillary loops in non-sclerotic glomeruli	Not established		
Interstitial fibrosis—in the cortical sections	Not established		
Tubular atrophy—in areas of cortical tubules	Not established		
Fiber-intimal arterial thickening with lumen reduction in areas covering this indicator	Not established		
Increase in mesangial matrix- in non-sclerotic glomeruli	Not established		
Presentation of foamy cells	Sporadic		

Legend: * The investigated slides contain more than 10 glomeruli for histological evaluation located adjacent to more than two arterial irrigation segments.

## Data Availability

Not applicable.

## Sample Availability

Samples of the tested extracts are available from the authors.
